# Synthesis and structure of *trans*-bis(1,4-dimesityl-3-methyl-1,2,3-triazol-5-ylidene)palladium(II) dichloride and diacetate. Suzuki–Miyaura coupling of polybromoarenes with high catalytic turnover efficiencies

**DOI:** 10.3762/bjoc.9.79

**Published:** 2013-04-10

**Authors:** Jeelani Basha Shaik, Venkatachalam Ramkumar, Babu Varghese, Sethuraman Sankararaman

**Affiliations:** 1 Department of Chemistry, Indian Institute of Technology Madras, Chennai 600036, India; 2 Sophisticated Analytical Instrument Facility, Indian Institute of Technology Madras, Chennai 600036, India

**Keywords:** C–C coupling, *N*-heterocyclic carbene, palladium, Suzuki–Miyaura coupling, 1,2,3-triazolylidene

## Abstract

*trans*-Bis(1,4-dimesityl-3-methyl-1,2,3-triazol-5-ylidene)palladium(II) dichloride has been shown to be an excellent catalyst for the multiple Suzuki–Miyaura coupling reactions of polybromoarenes to the corresponding fully substituted polyarylarenes. The reactions proceeded in excellent yields and with high turnover numbers. With 1,4-dibromobenzene the catalyst was found to be active for up to 13 consecutive cycles with a turnover number of 1260. The polyarylarenes were obtained in pure form after crystallization once without recourse to chromatographic purification. The single-crystal X-ray structures of the chloro (**1**) as well as the corresponding acetato (**2**) complexes are also reported and compared with the corresponding complexes of 1,4-diphenyl-3-methyl-1,2,3-triazol-5-ylidene as the ligand.

## Introduction

Over the past decade *N*-heterocyclic carbenes (NHCs) have attracted the attention of synthetic and organometallic chemists tremendously [1–5]. NHCs have been proven to be useful as organocatalysts in organic synthesis [6–9]. They are excellent ligands for transition-metal and lanthanide metal chemistry [1,10–11]. Over the past few years they have gradually replaced the conventional phosphane ligands. The transition-metal complexes of these versatile ligands have been shown to be excellent catalysts for various organic transformations [9–14]. Among the various NHCs 1,3-diarylimidazolylidenes are the most widely studied systems [12–14]. In the past five years 1,2,3-triazol-5-ylidenes have emerged as promising ligands for transition-metal chemistry [15–20]. 1,2,3-Triazol-5-ylidenes have been termed as abnormal NHCs and mesoionic carbenes because their structures cannot be represented in neutral canonical form [17,19]. Mesoionic NHCs are stronger sigma donors than the normal NHCs (e.g., imidazolylidenes versus triazolylidenes) [21–23]. Hence metal complexes of mesoionic carbene ligands are expected to show high stability and exceptional catalytic properties in comparison with their normal NHC counterparts. In particular triazolylidenes with sterically demanding mesityl and 2,4,6-triisopropylphenyl wingtip groups are catalytically very active. Polyphenylated arenes are a very important class of compounds and they find application in the areas of molecular electronics, organic discotic liquid crystals and OLEDs [24–25]. One of the ways to approach the synthesis of these interesting compounds is to carry out multiple C–C coupling reactions with suitable polybromoarene derivatives. In multiple C–C coupling reactions one often encounters the formation of partially coupled products and incomplete reactions leading to problematic separation of pure fully substituted compounds. Herein we report very clean multiple Suzuki–Miyaura coupling of polybromoarenes. In every case reported herein the final product, namely fully substituted polyarylarenes, was isolated in pure form upon single crystallization of the crude product. These reactions also proceeded with very high turnover number. We also report the structures of palladium(II) dichloride complex **1** and diacetate complex **2**.

## Results and Discussion

### Synthesis of Pd complexes

**Figure 1:**
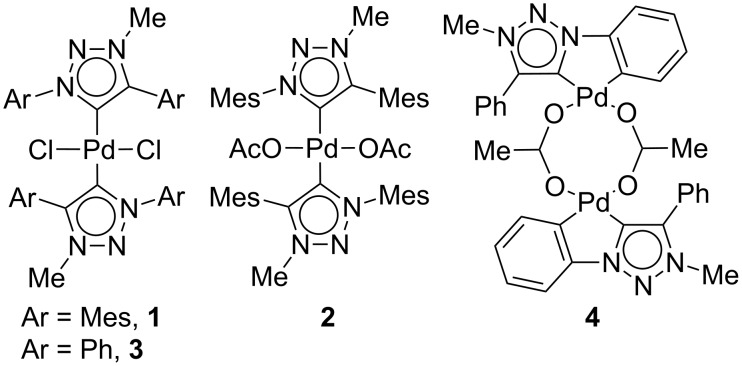
Structure of Pd complexes **1–4**.

**Scheme 1:**
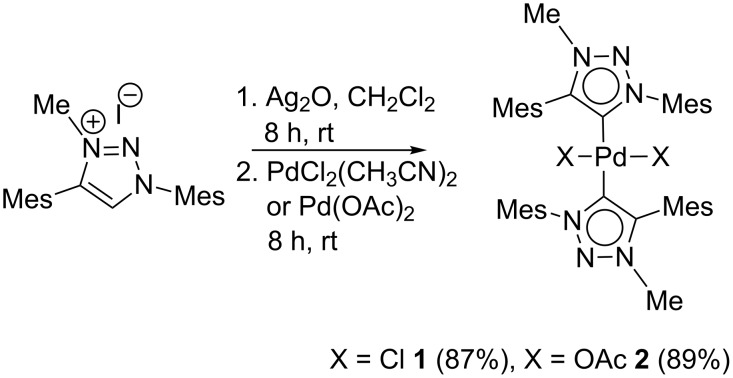
Synthesis of Pd complexes **1** and **2**.

Complexes **1** and **2** were synthesized from the corresponding silver carbene complex by transmetalation as reported earlier for the synthesis of bis(1,4-diphenyl-3-methyl-1,2,3-triazol-5-ylidene)palladium(II) derivatives **3** and **4** (Figure 1 and Scheme 1), respectively [26–27]. Treatment of 1,4-dimesityl-3-methyl-1,2,3-triazolium iodide with freshly prepared silver oxide followed by transmetalation with Pd(Cl)_2_(CH_3_CN)_2_ yielded **1** as a pale yellow solid in 87% (Scheme 1). The acetate complex **2** was prepared by the transmetalation of the silver carbene complex with Pd(OAc)_2_. Addition of Pd(OAc)_2_ to in situ generated silver carbene complex yielded **2** in 89% as a pale yellow solid (Scheme 1).

### Crystal structure of complex **1** and **2**

**Figure 2:**
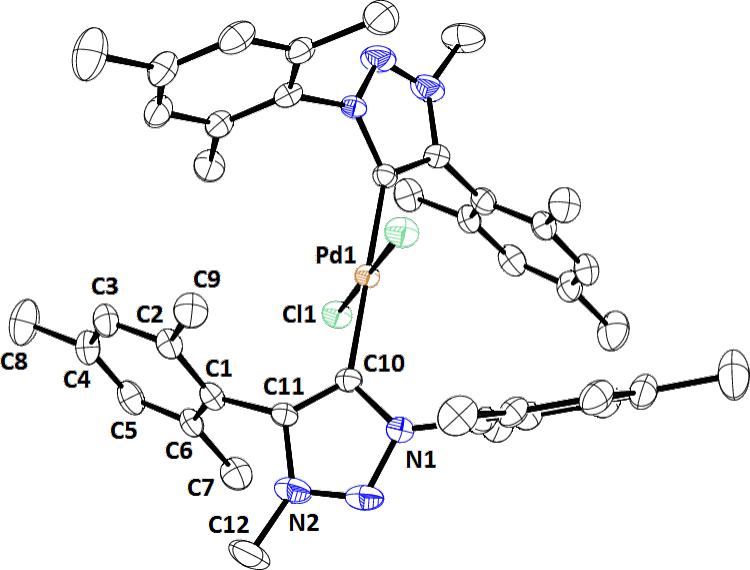
ORTEP representation of the structure of complex **1** in the crystal (35% probability ellipsoids). Hydrogens are omitted for clarity.

Although the synthesis of **1** was reported by Fukuzawa [27] the crystal structure of this complex was not reported. Slow evaporation of an acetonitrile solution of **1** yielded single crystals suitable for X-ray crystallography. Complex **1** crystallized in the tetragonal space group *I*4_1_/*acd*. The structure of the complex in the crystal clearly showed it to be a mononuclear complex with two chloro ligands *trans* with respect to each other, the two triazolylidene ligands also *trans* with respect to each other, and with the palladium in square planar geometry (Figure 2). The structures of **1** and **3** revealed that the Pd–C and Pd–Cl distances are comparable in both complexes. However the dihedral angle between the planes containing each of the triazolylidene rings in **3** is zero, i.e., they lie on parallel planes with the distance between the planes being 0.305 Å [26]. In the case of **1** the dihedral angle between the planes is 55.47°. In order to accommodate the bulky mesityl wingtip groups in **1** the carbene ligands are twisted with respect to the C_carbene_–Pd–C_carbene_ axis making a dihedral angle of 55.47°. The mesityl rings are also significantly twisted out of plane with respect to the triazolylidene rings in **1** compared to the twist of the phenyl rings in **3**.

**Figure 3:**
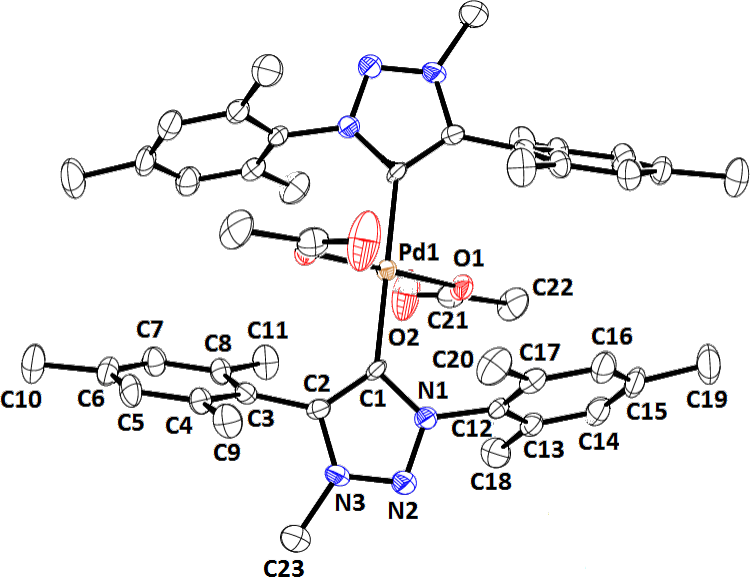
ORTEP representation of the structure of complex **2** in the crystal (35% probability ellipsoids). Hydrogens are omitted for clarity.

The structure of **2** was also unambiguously established by single-crystal XRD data. Crystals suitable for XRD were grown by slow evaporation of a toluene solution of **2**. It belonged to a monoclinic system with the space group *P*2_1_/*n*. The structures of the acetate complexes **2** and **4** are significantly different. Complex **4** is binuclear with each of the palladium atoms being part of a pallado cycle formed by insertion to the *ortho* position of one of the *N*-phenyl groups [26]. The two palladium atoms are connected by two bridging acetate ligands. In the case of **2** with dimesityl wingtip groups on the triazolylidene ligand the formation of a pallado cycle is not feasible because the *ortho* positions are substituted with methyl groups. Secondly, due to the bulky nature of the mesityl groups, the formation of a binuclear complex with bridging acetate ligands is also infeasible. Complex **2** has a simple mononuclear structure with two monodentate acetate ligands each bonded to palladium through a single oxygen atom (Figure 3).

### Suzuki–Miyaura coupling of polybromoarenes using complex **1**

**Scheme 2:**
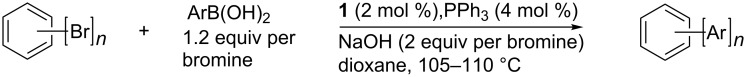
Multiple Suzuki–Miyaura coupling of polybromoarenes using complex **1**.

Complexes of palladium-mesoionic NHCs have been shown to be catalytically much more active than the corresponding complexes with normal NHCs (i.e., Pd complexes of 1,2,3-triazolylidene versus imidazolylidene ligands) in C–C bond-forming coupling reactions [28]. The enhanced catalytic activity may be due to stronger sigma donor ability of mesoionic NHCs compared to normal NHCs. The stronger sigma donor ability of mesoionic NHCs may stabilize intermediates in the catalytic cycle. Complex **1** has been shown by Fukuzawa [27] to be very active for the coupling of simple aryl chlorides. In this work we demonstrate that complex **1** is an excellent catalyst for multiple Suzuki–Miyaura coupling reactions of polybromoarenes. Reaction conditions were optimized using 1,4-dibromobenzene (**6**) and phenylboronic acid (**5**) as substrates. The reactions were carried out by using 2 mol % of complex **1** as catalyst and 4 mol % of PPh_3_ *irrespective of the number of bromines present in the polybromoarenes* (Scheme 2, Table 1). Typically for polybromoarenes 1.0 to 1.2 equivalents of arylboronic acid and 2 equivalents of NaOH *per bromine* were used. For example, in the case of 1,4-dibromobenzene (**6**), 2 equivalents of phenylboronic acid (**5**) and 4 equivalents of NaOH were used and in the case of 1,3,6,8-tetrabromopyrene (**18**) 4.8 equivalents of phenylboronic acid (**5**) and 8 equivalents of NaOH were used. The addition of PPh_3_ is crucial, and in its absence the reaction mixture turned black, the catalyst was quickly deactivated, and the reactions did not proceed to completion. The reactions were carried out at 105–110 °C under N_2_ atmosphere and the course of the reaction was followed by TLC. Typically, the reaction of polybromoarenes initially showed multiple spots on TLC. Reactions were carried out for the desired time period until a single major spot was observed on TLC. Pure polyarylated products were obtained by a single recrystallization of the crude product eliminating cumbersome chromatographic separations. Several polybromoarenes were investigated and the results are summarized in Table 1. In the case of **7** the catalytic activity was tested for up to 13 cycles by the successive addition of 1,4-dibromobenzene (**6**), phenylboronic acid (**5**) and NaOH to the same reaction pot. After 13 cycles of reaction, the catalytic turnover number (TON) was as high as 1260. TON is defined as the ratio of the number of moles of product formed to the number of moles of catalyst used times the number of C–C bonds formed in the reaction. This is due to the fact that for each C–C bond-forming reaction one catalytic cycle needs to be completed. It must be highlighted here that 2,7-di-*tert*-butyl-4,5,9,10-tetrabromopyrene (**21**) and 4,7,12,15-tetrabromo[2.2]paracyclophane (**26**) [29] are particularly difficult substrates to undergo Suzuki–Miyaura coupling in the presence of conventional catalysts such as PdCl_2_(PPh_3_)_2_, Pd(PPh_3_)_4_ and Pd(dba)_2_. The coupling of 4,7,12,15-tetrabromo[2.2]paracyclophane (**26**) with phenylmagnesium bromide in the presence of a NiCl_2_(PPh_3_)_2_ catalyst has been reported to yield 4,7,12,15-tetraphenyl[2.2]paracyclophane (**27**) in only 6% [29]. In the present study these substrates underwent four-fold Suzuki–Miyaura coupling smoothly resulting in the formation of fully substituted derivatives in near quantitative yields. Interestingly, hexabromobenzene (**16**) [30] and hexabromotriphenylene **23** [31] also underwent six-fold coupling in a clean manner resulting in the formation of hexaphenylbenzene (**17**) and hexaaryltriphenylene **24**, respectively, in good yields. Under these conditions polychloroarenes did not give a clean reaction, and the reactions were sluggish in comparison to polybromoarenes. In addition, unlike the polybromo derivatives, the polychloroarene derivatives of pyrene, triphenylene and [2,2]paracyclophane are not readily available.

**Table 1:** Multiple Suzuki–Miyaura coupling of polybromoarenes with arylboronic acids by using complex **1**^a^.

Entry	Polybromoarene	ArB(OH)_2_	Time (h)	Product (% Yield, TON^b^)

1	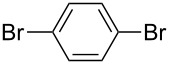 **6**	PhB(OH)_2_ (**5**)	3	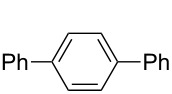 **7** (97, 1260 after 13 cycles^c^)
2	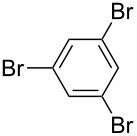 **9**	PhB(OH)_2_ (**5**) 4-CF_3_C_6_H_4_B(OH)_2_ (**8**)	3.5 3	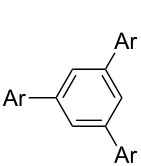 Ar = Ph, **10** (94, 141) Ar = 4-CF_3_C_6_H_4_, **11** (94, 141)
3	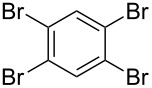 **12**	PhB(OH)_2_ (**5**)	9	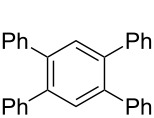 **13** (95, 193)
4	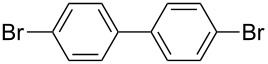 **14**	PhB(OH)_2_ (**5**)	7	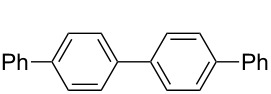 **15** (97, 194)
5	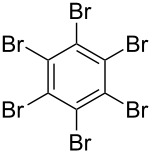 **16**	PhB(OH)_2_ (**5**)	15	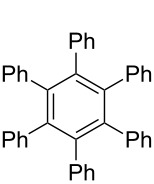 **17** (59, 178)
6	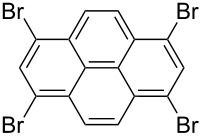 **18**	PhB(OH)_2_ (**5**) 4-CF_3_C_6_H_4_B(OH)_2_ (**8**)	8 6	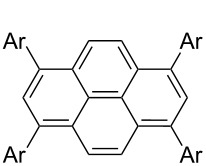 Ar = Ph, **19** (97, 194) Ar = 4-CF_3_C_6_H_4_, **20** (94, 188)
7	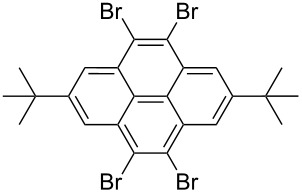 **21**	PhB(OH)_2_ (**5**)	9.5	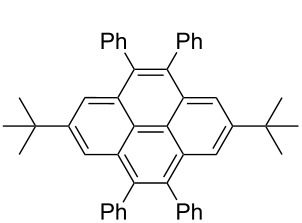 **22** (95, 190)
8	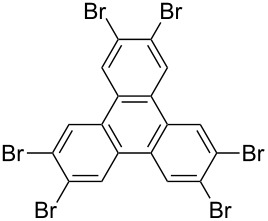 **23**	PhB(OH)_2_ (**5**) 4-CF_3_C_6_H_4_B(OH)_2_ (**8**)	7.5 7	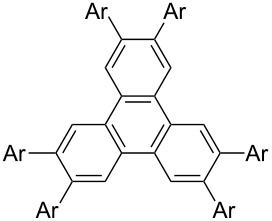 Ar = Ph, **24** (99, 297) Ar = 4-CF_3_C_6_H_4_, **25** (95, 285)
9	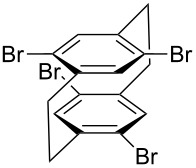 **26**	PhB(OH)_2_ (**5**) 4-CF_3_C_6_H_4_B(OH)_2_ (**8**)	10 10	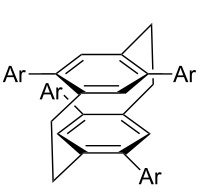 Ar = Ph, **27** (93, 187) Ar = 4-CF_3_C_6_H_4_, **28** (88, 176)

^a^100 mg scale bromoarene, 2 mol % catalyst **1**, 4 mol % PPh_3_, 1.2 equiv ArB(OH)_2_ per bromine atom, 2 equiv NaOH per bromine; ^b^TON = (moles of product formed × number of bromine atoms substituted)/(moles of catalyst used); ^c^substrates (100 mg bromoarene, 1.2 equiv ArB(OH)_2_ and 2 equiv NaOH) were added to the same pot up to 13 cycles after completion of the previous cycle.

## Conclusion

In conclusion, the dichloro complex **1** has been shown to be catalytically very active for multiple Suzuki–Miyaura coupling of polybromoarenes, and several otherwise difficult to treat polybromo substrates have been efficiently converted to the corresponding polyaryl derivatives by using this catalyst. We also report the structural characterization of dichloro (**1**) and diacetate (**2**) complexes. Comparison of the structures of **1** and **2** with that of the corresponding (1,4-diphenyl-3-methyl-1,2,3-triazol-5-ylidene)palladium(II) complexes **3** and **4** is presented. The structures of the diacetate complexes **2** and **4** are significantly different in that **4** is a cyclopalladated dinuclear complex with acetate bridges whereas **2** is a mononuclear complex with monodentate acetate ligands. We predict that these catalysts could be potentially useful in polymer chemistry for the synthesis of polyaryl polymers that are important in OLEDs and molecular electronics applications.

## Experimental

1,4-Dimesityl-3-methyl-1,2,3-triazolium iodide was prepared from the corresponding triazole, which in turn was prepared by the click reaction of mesitylacetylene and mesitylazide according to the literature [27]. Complex **1** was synthesized in a similar manner as reported previously [27].

### Synthesis of complex **1** [27]

1,4-Dimesityl-3-methyl-1,2,3-triazolium iodide (250 mg, 0.56 mmol) was treated with freshly prepared silver oxide (78 mg, 0.34 mmol, 0.6 equiv) in CH_2_Cl_2_ (12 mL). The solution was stirred at room temperature in the dark for 8 h. The silver carbene complex thus generated was not isolated. It was directly treated with Pd(CH_3_CN)_2_Cl_2_ (83 mg, 0.34 mmol, 0.6 equiv) and stirred for 8 h. The reaction mixture was passed through a bed of celite, and then removal of CH_2_Cl_2_ gave complex **1** as a pale yellow solid (396 mg, 10.49 mmol) in 87% yield. Crystals of **1** suitable for single-crystal diffraction were grown by slow evaporation of a solution of **1** in acetonitrile. Complex **1** decomposed at 258–260 °C without melting. ^1^H NMR (400 MHz, CDCl_3_) δ 6.95 (s, 8H) 3.72 (s, 6H), 2.45 and 2.44 (two overlapping singlets, 12H), 1.98 and 1.97 (two overlapping singlets, 24H); ^13^C NMR (100 MHz, CDCl_3_) δ 162.8 (*C*_carbene_), 143.8, 139.7, 139.0, 138.8, 136.1, 135.9, 128.8, 128.3, 124.3, 35.7, 21.6, 21.5, 21.1, 18.9; ESIMS: *m*/*z* 815 along with the expected isotope peaks. HRMS (ESI–QTOF): *m*/*z* calcd for C_42_H_50_N_6_Cl_2_Pd 815.2587, found 815.2574.

### Synthesis of complex **2**

The silver carbene complex generated as described above was subsequently treated with Pd(OAc)_2_ (75 mg, 0.34 mmol, 0.6 equiv) and stirred for 8 h. The reaction mixture was passed through a bed of celite, and then dichloromethane was removed under vacuum to give complex **2** as a pale yellow solid (430 mg, 0.5 mmol) in 89%.Crystals of **2** suitable for single crystal diffraction were grown by slow evaporation of a solution of **2** in toluene. Complex **2** decomposed at 149–152 °C without melting. ^1^H NMR (400 MHz, CDCl_3_) δ 7.07–6.87 (m, 8H), 3.85–3.68 (m, 6H), 2.42–2.40 (m, 12H), 2.14–2.13 (m, 6H), 2.04–1.96 (m, 18H), 1.25 (s, 6H); ^13^C NMR (100 MHz, CDCl_3_) δ 181.5, 162.8, 143.7, 140.9, 140.7, 139.6, 139.39, 139.3, 138.8, 138.7, 135.8, 135.7, 135.2, 135.0, 130.0, 129.4, 129.0, 128.7, 128.2, 36.4, 35.8, 35.7, 23.3, 21.5, 21.48, 21.43, 21.0, 20.0, 18.8, 17.4; ESIMS: *m*/*z* 863 along with the expected isotope peaks. HRMS (ESI–QTOF): *m*/*z* calcd for C_46_H_56_N_6_O_4_Pd 863.3476, found 863.3491.

### Representative procedures for Suzuki–Miyaura coupling of polybromoarenes:

**Synthesis of 1,3,6,8-tetraphenylpyrene (19):** A mixture of 1,3,6,8-tetrabromopyrene (**18**, 100 mg, 0.193 mmol), phenylboronic acid (**5**, 113 mg, 0.93 mmol, 4.8 equiv), complex **1** (3 mg, 2 mol %), triphenylphosphine (4 mg, 4 mol %), NaOH (62 mg, 1.54 mmol, 8 equiv) was heated under reflux in 4 mL of 1,4-dioxane under nitrogen atmosphere. The reaction was completed in 8 h (monitored by TLC). The reaction mixture was diluted with water (10 mL) and extracted with CH_2_Cl_2_ (3 × 5 mL). The organic layer was dried over sodium sulfate and filtered, and then the solvent was removed under vacuum. The crude product was recrystallized from CH_2_Cl_2_ to give 1,3,6,8-tetraphenylpyrene (**19**, 95 mg, 0.19 mmol) in 97% yield as a lime-yellow solid, mp: 264 °C. ^1^H NMR (400 MHz, CDCl_3_) δ 8.17 (s, 4H), 8.01 (s, 2H), 7.68–7.66 (m, 8H), 7.55–7.52 (m, 8H), 7.48–7.44 (m, 4H); ^13^C NMR (100 MHz, CDCl_3_) δ 141.2, 137.4, 130.8, 129.6, 128.5, 128.2, 127.4, 126.1, 125.4; HRMS (ESI–QTOF): *m*/*z* calcd for C_40_H_27_ 507.2113, found 507.2094.

**1,3,6,8-Tetrakis(4-trifluoromethylphenyl)pyrene (20)**: Prepared from 1,3,6,8-tetrabromopyrene (**18**, 100 mg, 0.19 mmol), complex **1** (3 mg, 2 mol %), 4-trifluoromethylphenylboronic acid (**8**, 176 mg, 0.93 mmol), NaOH (62 mg, 1.54 mmol), PPh_3_ (2 mg, 4 mol %). Yield 142 mg, 94%; colorless solid, mp 231–232 °C (lit. 231 °C) [32]. ^1^H NMR (500 MHz, CDCl_3_) δ 8.13 (s, 4H), 7.99 (s, 2H), 7.83–7.77 (AA’BB’ pattern, 16H); ^13^C NMR (125 MHz, CDCl_3_) δ 144.3, 136.4, 131.0, 130.0 (q, *J* = 31.8 Hz), 129.4, 128.5, 125.9, 125.7, 125.6 (q, *J* = 2.5 Hz), 124.4 (q, *J* = 270 Hz); HRMS (ESI–QTOF): *m*/*z* calcd for C_44_H_23_F_12_ 779.1608, found 779.1616.

**2,3,6,7,10,11-Hexakis(4-trifluoromethylphenyl)triphenylene (25)**: Prepared from 2,3,6,7,10,11-hexabromotriphenylene (**23**, 100 mg, 0.14 mmol), complex **1** (2 mg, 2 mol %), 4-trifluoromethylphenylboronic acid (**8**, 195 mg, 1.03 mmol), NaOH (68 mg, 1.70 mmol), PPh_3_ (2 mg, 4 mol %). Yield 148 mg, 95%; silver colored solid. (mp > 360 °C). ^1^H NMR (500 MHz, CDCl_3_) δ 8.70 (s, 6H), 7.60–7.43 (AA’BB’, 24H, *J* = 8 Hz); ^13^C NMR (125 MHz, CDCl_3_) δ 144.3, 139.2, 130.4, 129.7 (q, *J* = 32.5 Hz), 129.5, 126.1, 125.5 (q, *J* = 3.6 Hz), 124.2 (q, *J* = 270 Hz); HRMS (ESI–QTOF): *m*/z calcd for C_60_H_31_F_18_ 1093.2138, found 1093.2100.

**4,7,12,15-Tetrakis(4-trifluoromethylphenyl)[2.2]paracyclophane (28)**: Prepared from 4,7,12,15-tetrabromo[2.2]paracyclophane (**26**, 100 mg, 0.19 mmol), complex **1** (3 mg, 2 mol %), 4-trifluoromethylphenylboronic acid (**8**, 174 mg, 0.92 mmol), NaOH (61 mg, 1.53 mmol), PPh_3_ (2 mg, 4 mol %). Yield 132 mg, 88%; colorless solid, mp 224 °C. ^1^H NMR (500 MHz, CDCl_3_) δ 7.69–7.67 (m, 8H), 7.49–7.47 (m, 8H), 6.86 (s, 4H); 3.52 and 2.83 (AA’BB’ pattern, 8H); ^13^C NMR (125 MHz, CDCl_3_) δ 143.7, 139.5, 137.5, 132.6, 129.4 (q, *J* = 32.6 Hz), 129.2, 124.3 (q, *J* = 270 Hz), 33.4; HRMS (ESI–QTOF): *m*/*z* calcd for C_44_H_29_F_12_ 785.2078, found 785.2077.

## Supporting Information

File 1 Spectroscopic characterization data of compounds **8, 10, 11, 13, 15, 17, 22, 24** and **27**.

File 2 CIF file for complex **1**.

File 3 CIF file for complex **2**.
