# Supplementary material for: Synthesis and structure of trans-bis(1,4-dimesityl-3-methyl-1,2,3-triazol-5-ylidene)palladium(II) dichloride and diacetate. Suzuki–Miyaura coupling of polybromoarenes with high catalytic turnover efficiencies
Source: Beilstein J Org Chem. 2013 Apr 10;9:698–704. doi: 10.3762/bjoc.9.79 (PMC3628290; doi:10.3762/bjoc.9.79)
Supplement: File 1 — Spectroscopic characterization data of compounds 8, 10, 11, 13, 15, 17, 22, 24 and 27. [file Beilstein_J_Org_Chem-09-698-s001.pdf]

Supporting Information

for

Synthesis and structure of *trans*-bis(1,4-dimesityl-3-methyl-1,2,3-triazol-5-ylidene)palladium (II) dichloride and diacetate. Suzuki–Miyaura coupling of polybromoarenes with high catalytic turnover efficiencies

Jeelani Basha Shaik[1] , Venkatachalam Ramkumar[1], Babu Varghese[2] and Sethuraman Sankararaman*[1]

Address: [1]Department of Chemistry, Indian Institute of Technology Madras, Chennai 600036, India and [2] Sophisticated Analytical Instrument Facility, Indian Institute of Technology Madras, Chennai 600036, India.

Email: Sethuraman Sankararaman - sanka@iitm.ac.in

*Corresponding author

Spectroscopic characterization data of compounds 8, 10, 11, 13, 15, 17, 22, 24 and 27.

***p*-Terphenyl (7)**: Prepared from *p*-dibromobenzene (**6**, 100 mg, 0.42 mmol), complex **1** (7 mg, 2 mol %), phenylboronic acid (**5**) (124 mg, 1.02 mmol), NaOH (68 mg, 1.69 mmol) PPh_3 (5 mg, 4 mol %). Yield 95 mg, 97%; glittering white solid, mp 214 °C (lit 213–214 °C) [1]; ^1H NMR (400 MHz, $CDCl_3$) δ 7.68 (s, 4H), 7.66–7.64 (m, 4H), 7.48–7.44 (m, 4H), 7.38–7.34 (m, 2H); ^{13}C NMR (100 MHz, $CDCl_3$) δ 140.8, 140.2, 128.9, 127.6, 127.4, 127.2.

1,3,5-Triphenylbenzene (10): Prepared from 1,3,5-tribromobenzene (**9**, 100 mg, 0.38 mmol), complex **1** (5 mg, 2 mol %), phenylboronic acid (**5**, 139 mg, 1.43 mmol), NaOH (76 mg, 1.9 mmol) PPh_3 (3 mg, 4 mol %). Yield 92 mg, 94%; colorless crystals, mp 176–178 °C (lit 174–175 °C) [2]; ^1H NMR (400 MHz, $CDCl_3$) δ 7.80 (s, 3H), 7.73–7.70 (m, 6H), 7.51–7.48 (m, 6H), 7.42–7.38 (m, 3H); ^{13}C NMR (100 MHz, $CDCl_3$) δ 142.4, 141.2, 128.9, 127.6, 127.5, 125.3.

1,3,5-Tris-(4-trifluoromethylphenyl)benzene (11): Prepared from 1,3,5-tribromobenzene (**9**, 100 mg, 0.32 mmol), complex **1** (5 mg, 2 mol %), 4-trifluoromethylphenylboronic acid (**8**, 217 mg, 1.45 mmol), NaOH (76 mg, 1.9 mmol), PPh_3 (3 mg, 4 mol %); mp 231 °C (lit 231 °C) [3]; yield 153 mg, 94%; glittering light yellow solid, mp 231 °C. ^1H NMR (500 MHz, $CDCl_3$) δ 7.83 (s, 3H), 7.82–7.75 (AA'BB' pattern, 12H); ^{13}C NMR (125 MHz, $CDCl_3$) δ 144.1, 141.6, 130.2 (q, J = 32.4 Hz), 127.8, 127.5, 126.3, 126.1 (q, J = 3.75 Hz), 124.3 (q, J = 270 Hz).

1,2,4,5-Tetraphenylbenzene (13): Prepared from 1,2,4,5-tetrabromobenzene (**12**, 100 mg, 0.25 mmol), complex **1** (4 mg, 2 mol %), phenylboronic acid (**5**, 186 mg, 1.52 mmol), NaOH (81 mg, 2.03 mmol) PPh_3 (3 mg, 4 mol %). Yield 93 mg, 95%; colorless solid, mp 269 °C (lit 267–268 °C) [4]. ^1H NMR (400 MHz, $CDCl_3$) δ 7.53 (s, 2H), 7.23 (s, broad, 20H); ^{13}C NMR (100 MHz, $CDCl_3$) δ 141.1, 139.7, 133.1, 130.0, 128.1, 126.7.

***p*-Quaterphenyl (15)**: Prepared from 4,4'-dibromobiphenyl (**14**, 100 mg, 0.32 mmol), complex **1** (5 mg, 2 mol %), phenylboronic acid (**5**, 94 mg, 0.77 mmol), NaOH (51 mg, 1.28 mmol) PPh_3 (4 mg, 4 mol %). Yield 96 mg, 97%; glittering white solid, mp 322 °C (lit 322 °C) [5]; ^1H NMR

(500 MHz, $CDCl_3$) δ 7.74 and 7.70 (AB quartet, J = 8.5 Hz, 8H), 7.67–7.66 (m, 4H), 7.48–7.45 (m, 3H), 7.38–7.35 (m, 3H); ^{13}C NMR (125 MHz, $CDCl_3$) δ 144.6, 140.8, 140.3, 139.7, 128.9, 127.7, 127.5, 127.1.

Hexaphenylbenzene (17) [6,7]: Prepared from hexabromobenzene (**16**, 100 mg, 0.18 mmol), complex **1** (3 mg, 2 mol %), phenylboronic acid (**5**, 159 mg, 1.31 mmol), NaOH (87 mg, 2.18 mmol) PPh_3 (2 mg, 4 mol %). Yield 58 mg, 59%; colorless solid, mp > 360 °C; ^1H NMR (400 MHz, $CDCl_3$) δ 6.85–6.81 (m, 30H); ^{13}C NMR (100 MHz, $CDCl_3$) δ 140.7, 140.4, 131.5, 126.7, 125.3.

2,7-Di-*tert*-butyl-4,5,9,10-tetraphenylpyrene (22): Prepared from 2,7-di-*tert*-butyl-4,5,9,10-tetrabromopyrene (**21**, 100 mg, 0.18 mmol), complex **1** (3 mg, 2 mol %), phenylboronic acid (**5**, 159 mg, 1.31 mmol), NaOH (87 mg, 2.18 mmol) PPh_3 (2 mg, 4 mol %). Yield 110 mg, 95%; colorless solid, mp 355–357 °C. ^1H NMR (400 MHz, $CDCl_3$) δ 7.86 (s, 4H), 7.30–7.24 (m, 20H), 1.23 (s, 10H); ^{13}C NMR (100 MHz, $CDCl_3$) δ 148.3, 140.0, 137.9, 131.3, 130.9, 127.7, 126.6, 122.1, 35.4, 31.7; HRMS (ESI–QTOF): m/z calcd for $C_{48}H_{43}$ 619.3365, found 619.3350.

2,3,6,7,10,11-Hexaphenyltriphenylene (24): Prepared from 2,3,6,7,10,11-hexabromotriphenylene (**23**, 100 mg, 0.19 mmol), complex **1** (2 mg, 2 mol %), phenylboronic acid (**5**, 156 mg, 1.28 mmol), NaOH (68 mg, 1.71 mmol), PPh_3 (2 mg, 4 mol %). Yield 96 mg, 99%; white solid, mp 352 °C. ^1H NMR (400 MHz, $CDCl_3$) δ 8.71 (s, 6H), 7.34–7.27 (m, 30H); ^{13}C NMR (100 MHz, $CDCl_3$) δ 141.6, 140.1, 130.2, 129.1, 128.1, 126.9, 125.7; HRMS (ESI–QTOF): m/z calcd for $C_{54}H_{37}$ 685.2895, found 685.2918.

4,7,12,15-Tetraphenyl[2.2]paracyclophane (27) [8]: Prepared from 4,7,12,15-tetrabromo[2.2]paracyclophane (**26**, 100 mg, 0.19 mmol), complex **1** (3 mg, 2 mol %), phenylboronic acid (**5**, 112 mg, 0.92 mmol), NaOH (61 mg, 1.53 mmol) PPh_3 (2 mg, 4 mol %). Yield 91 mg, 93%; colorless solid, mp 208–210 °C. ^1H NMR (400 MHz, $CDCl_3$) δ 7.42–7.31 (m,

20H), 6.86 (s, 4H), 3.57–3.49 and 2.84–2.76 (m, AA'BB' pattern, 4H); ^{13}C NMR (100 MHz, $CDCl_3$) δ 140.8, 140.0, 137.1, 132.5, 129.2, 128.6, 126.8, 33.5.

References

1. Planellas, M.; Pleixats, R.; Shafir, A. *Adv. Synth. Catal.* **2012,** *354,* 651–662. doi:10.1002/adsc.201100574
2. Bai, L.; Wang, J. *Adv. Synth. Catal.* **2008,** *350,* 315–320. doi:10.1002/adsc.200700361
3. Iannazzo, L.; Vollhardt, K. P. C.; Malacria, M.; Aubert, C.; Gandon, V. *Eur. J. Org. Chem.* **2011,** 3283–3292. doi:10.1002/ejoc.201100371
4. Harada, K.; Hart, H.; Frank Du, C. *J. Org. Chem.* **1985,** *50,* 5524–5528. doi:10.1021/jo00350a018
5. Vajpayee, V.; Song, Y. H.; Ahn, J. S.; Chi, K. *Bull. Korean Chem. Soc.* **2011,** *32,* 2970–2972. doi:10.5012/bkcs.2011.32.8.2970
6. Newkome, G. R.; Islam, N. B.; Robinson, M. J. *J. Org. Chem.* **1975,** *40,* 3514–3518. doi:10.1021/jo00912a010
7. Tu, T.; Sun, Z.; Fang, W.; Xu, M.; Zhou, Y. *Org. Lett.* **2012,** *14,* 4250–4253. doi:10.1021/ol3019665
8. König, B.; Knieriem, B.; de Meijere, A. *Chem. Ber.* **1993,** *126,* 1643–1650.
